# Ventricular Septal Perforation Associated with Takotsubo Syndrome and Its Pathogenic Mechanisms

**DOI:** 10.70352/scrj.cr.25-0719

**Published:** 2026-02-03

**Authors:** Masaru Yoshikai, Takahiro Miho, Kiyokazu Koga, Nozomi Yoshida, Naoyo Nishida

**Affiliations:** 1 Department of Cardiovascular Surgery, Shin-Koga Hospital, Kurume, Fukuoka, Japan; 2 Department of Pathology, Shin-Koga Hospital, Kurume, Fukuoka, Japan

**Keywords:** ventricular septal perforation, Takotsubo syndrome, contraction band necrosis, left ventricular wall tension, Laplace’s law

## Abstract

**INTRODUCTION:**

Takotsubo syndrome (TTS), once considered a benign self-limiting disease, is now recognized to have substantial short-term morbidity and mortality. During the acute phase, it can result in severe heart failure, cardiogenic shock, arrhythmias, cardiac rupture, ventricular septal perforation (VSP), and thromboembolic events, with in-hospital mortality rates comparable to those observed in acute coronary syndrome. Although VSP is rare, it represents a potentially lethal complication of TTS. Herein, we present a case of VSP associated with TTS and discuss a mechanism for the development of VSP in TTS.

**CASE PRESENTATION:**

A 69-year-old female presented with dyspnea. The diagnosis of TTS was established based on the presence of apical ballooning of the left ventricle (LV), hyperkinesis of the LV basal walls, normal coronary arteries, and apical defect in myocardial scintigraphy. Additionally, a VSP was identified, and given the stabilization of hemodynamics with an intra-aortic balloon pump and medical treatment, it was deemed preferable to delay surgical treatment. Approximately 1 month later, after the LV wall motion abnormality had been normalized, the VSP was successfully closed. Histopathological examination of the ventricular septum revealed contraction band necrosis along with fibrosis and focal cardiomyocyte necrosis.

**CONCLUSIONS:**

While VSP is a potentially lethal complication of TTS, recovery of LV function can be anticipated, suggesting that surgical treatment could be deferred, provided that hemodynamic stability is maintained. This case demonstrates that focal myocardial necrosis can occur in TTS. Furthermore, increased wall tension occurs in the ballooning segments during systole, as the LV radius increases and the ventricular wall fails to thicken, in accordance with Laplace’s law. Both myocardial fragility resulting from focal myocardial necrosis and increased LV wall tension due to systolic ballooning are considered to be key mechanisms underlying the development of VSP in TTS.

## Abbreviations

ACS
: acute coronary syndrome
CBN
: contraction band necrosis
ECG
: electrocardiogram
I-123 BMIPP
: Iodine-123 beta-methyl-p-iodophenyl-pentadecanoic acid
IABP
: intra-aortic balloon pump
LV
: left ventricle
LVOTO
: left ventricular outflow tract obstruction
MR
: mitral regurgitation
Qp/Qs
: pulmonary-to-systemic blood flow ratio
TR
: tricuspid regurgitation
TTE
: transthoracic echocardiography
TTS
: Takotsubo syndrome
VSP
: ventricular septal perforation

## INTRODUCTION

TTS, once considered a benign self-limiting disease, is now recognized to have substantial short-term morbidity and mortality.^1–3)^ During the acute phase, it can lead to severe heart failure, cardiogenic shock, arrhythmias, cardiac rupture, VSP, and thromboembolic events,^1,2)^ with hospital mortality rates comparable to those of ACS.^1,3)^ While VSP is rare, it represents a potentially lethal complication of TTS. Herein, we present a case of VSP associated with TTS and discuss mechanisms for the development of VSP in TTS.

## CASE PRESENTATION

**Fig. 1:**
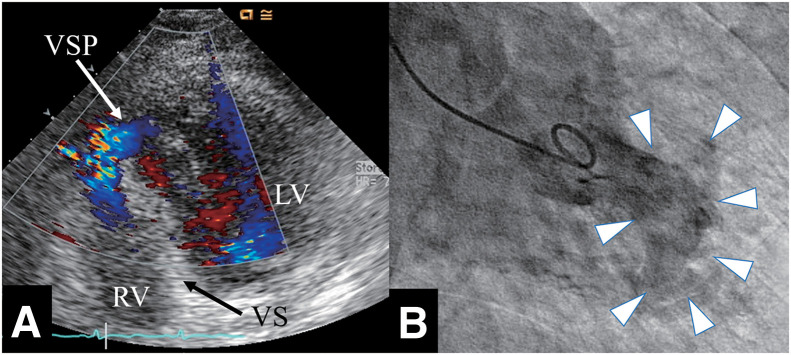
Transthoracic echocardiography and left ventriculography findings. Echocardiography reveals ventricular septal perforation at the apical ventricular septum (**A**). Left ventriculography shows ballooning of the left ventricular apical wall (arrowheads, **B**). LV, left ventricle; RV, right ventricle; VS, ventricular septum; VSP, ventricular septal perforation

**Fig. 2:**
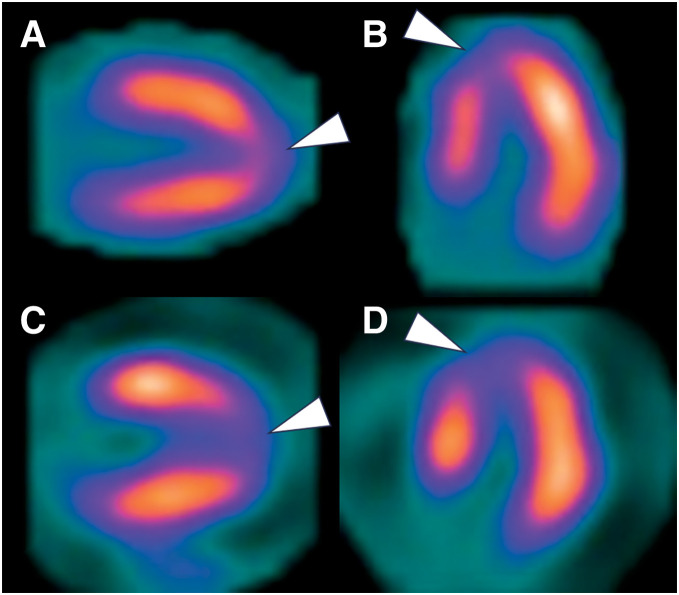
Myocardial scintigraphy findings. Myocardial scintigraphy with Thallium-201 (**A**, **B**) and I-123 BMIPP (**C**, **D**) revealed an apical defect (arrowheads), with the defect being more pronounced in the I-123 BMIPP images. I-123 BMIPP, Iodine-123 beta-methyl-p-iodophenyl-pentadecanoic acid

**Fig. 3:**
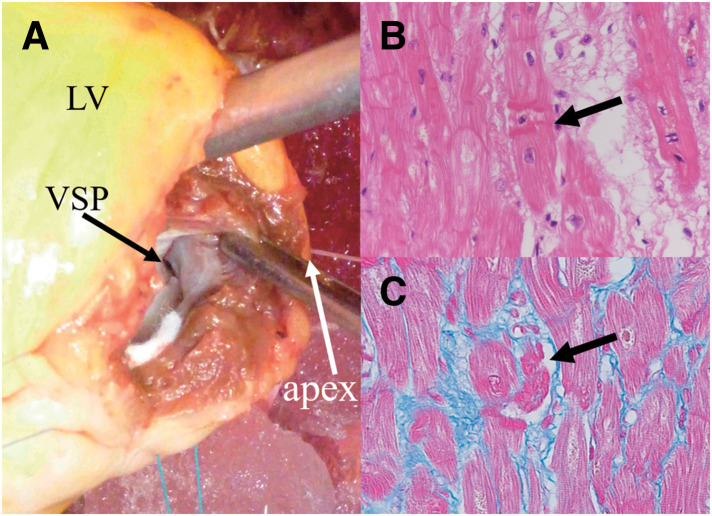
Operative and histopathological findings. Operative finding shows a ventricular septal perforation at the apical ventricular septum (**A**). Histopathological examination of the ventricular septum revealed contraction band necrosis (Hematoxylin and eosin staining, 400× magnification) (**B**) along with fibrosis (stained blue) and focal cardiomyocyte necrosis (Azan staining, 400× magnification) (**C**). LV, left ventricle; VSP, ventricular septal perforation

A 69-year-old female presented to a local clinic with dyspnea that had developed over the past 3 days. A chest X-ray revealed pulmonary congestion and bilateral pleural effusion, leading to her admission with a diagnosis of heart failure. There were no notable events involving psychological or physical stress prior to the presentation. Laboratory findings showed elevated levels of LDH at 219 U/L and NT-proBNP at 3180 pg/mL, while CPK, GOT, GPT were within normal ranges, and CRP was negative. Troponin was not measured. The ECG displayed ST elevation in leads V2-V5 and terminal T wave inversion in V3-V6. TTE did not reveal LV enlargement or wall motion abnormalities, but estimated systolic pulmonary artery pressure was elevated at 55 mmHg, with moderate MR and severe TR noted. Two days later, giant negative T waves appeared in leads V3-V6 on the ECG, and TTE showed hypokinesis at the LV apex, prompting transfer to our hospital for heart failure treatment. Upon transfer, her blood pressure was 115/75 mmHg, pulse 93 bpm, and SpO2 was 96% on room air. A Levine III/VI pansystolic murmur was most prominent at the apex. Mild lower limb edema was observed, but no lung crackles were heard. Chest X-ray showed a cardiothoracic ratio of 55.2% with pulmonary congestion and bilateral pleural effusion. The ECG showed sinus rhythm at 80 bpm with negative T waves in leads I, II, aVL, aVF, V2, and giant negative T waves in V3-V6. TTE revealed LV end-diastolic/systolic dimensions of 49/27 mm, ventricular septal/posterior wall thickness of 9/9 mm, and an LV ejection fraction of 71%. Dyskinesis was observed in the apical septum and in the apical portions of the anterior, inferior, and lateral walls. Additionally, a VSP was observed at the apical ventricular septum (**Fig. 1A**) with a Qp/Qs of 2.0 and a left-to-right shunt ratio of 50%. Mild MR, moderate TR, bilateral pleural effusion, and a small amount of pericardial effusion were present, with an estimated pulmonary artery systolic pressure of 36 mmHg. No LVOTO was observed. Cardiac catheterization showed mean right atrial pressure of 13 mmHg, pulmonary artery pressure of 47/26 (31) mmHg, mean pulmonary artery wedge pressure of 20 mmHg, LV end-diastolic pressure of 33 mmHg, and a cardiac index of 1.84 L/min/m^2^. An O_2_ step-up was noted in the right ventricle, with the Qp/Qs of 2.2 and the left-to-right shunt ratio of 54.4%. Left ventriculography showed akinesis of the LV apex and hypercontraction at the LV base (**Fig. 1B**), with opacification of the right ventricle and pulmonary artery. Coronary angiography revealed no stenotic lesions, leading to the diagnosis of TTS. An IABP was inserted, and pharmacological therapy with diuretics (furosemide and spironolactone) and carperitide was initiated. Over the subsequent 2 days, a total urine output of 4100 mL was achieved, and the pulmonary artery pressure decreased to 22/4 (10) mmHg. Both the Qp/Qs and the left-to-right shunt ratio remained unchanged, at 2.0% and 49.3%, respectively. Chest X-ray demonstrated improvement in pulmonary congestion, accompanied by alleviation of heart failure symptoms. Consequently, the patient was successfully weaned from IABP support on day 3. MRI showed ballooning of the LV apex and hyperkinetic motion at the LV base. Myocardial scintigraphy using I-123 BMIPP and Thallium-201 revealed an apical defect, with the defect being more pronounced in the I-123 BMIPP images (**Fig. 2**), further supporting the diagnosis of TTS. Subsequently, heart failure symptoms resolved; however, preoperative TTE demonstrated no reduction in shunt severity, with a Qp/Qs of 1.9 and a left-to-right shunt ratio of 47.4%. The estimated pulmonary artery systolic pressure was 36 mmHg, and moderate MR and moderate TR persisted. In contrast, LV wall motion abnormalities had completely resolved. After careful consideration of these findings by the heart team, surgical intervention was deemed appropriate to prevent long-term cardiac dilatation, deterioration of cardiac function, and the development of heart failure. Surgery was performed on day 37 under cardiopulmonary bypass and cardiac arrest. An LV incision showed a VSP measuring 10 × 3 mm at the apical ventricular septum, surrounded by fibrotic tissue (**Fig. 3A**), and closed using a bovine pericardial patch sutured on the LV side. The LV incision was reinforced with felt strips and securely closed with double-layer sutures. The postoperative course was uneventful, and although mild hypokinesis was observed at the site of the LV incision, no residual shunt was detected on TTE; additionally, MR had resolved, and TR was reduced to a mild degree. Histopathological examination of the ventricular septum revealed CBN (**Fig. 3B**) along with focal cardiomyocyte necrosis and fibrosis (**Fig. 3C**). The patient was discharged on POD 12 and has remained in good health for 10 years after surgery, without any recurrence of TTS or heart failure.

## DISCUSSION

We herein present a case of VSP associated with TTS. Contrary to previous beliefs that TTS is a benign self-limiting disease, it is now recognized to involve substantial short-term morbidity and mortality.^1–3)^ During the acute phase, nearly half of patients manifest severe cardiac complications, including acute heart failure, cardiogenic shock, arrhythmias, LVOTO, MR, intracardiac thrombi, LV rupture, and VSP.^2)^ Conditions like acute heart failure, cardiogenic shock, arrhythmias, LV rupture, and VSP are potentially fatal, and the overall in-hospital mortality of TTS has been reported at 4.2%–4.5%,^2)^ comparable to those of ACS.^1,3)^ LV rupture and VSP are rare, occurring in less than 1% of cases,^1,2)^ with VSP being less frequent than LV rupture.^4)^ Including the present case, a search in the PubMed for English-language reports on VSP/ventricular septal rupture associated with TTS up to 2024 revealed 25 cases, of which all but one^5)^ involved the apical type of TTS, with the single remaining case classified as the midventricular type.^5)^ Notably, in both TTS subtypes, VSP consistently occurred within the ballooning segment. Among these, acute-phase mortality was 50% (4/8) with medical treatment, 0% (0/3) with transcatheter closure, and 28.6% (4/14) with surgical intervention, yielding an overall rate of 32%. Medical management alone often leads to rapid deterioration, with approximately half of patients dying within a few days.^6)^ Conversely, a few patients survive the acute phase with medical therapy. Therefore, as with VSP following ACS, surgical intervention is generally recommended.^2)^ Although VSP in both post-ACS and TTS-related settings results from rupture of the necrotic myocardium, the underlying pathophysiology differs between the 2 conditions. In post-ACS VSP, myocardial necrosis typically involves the territory perfused by the culprit coronary artery. In contrast, the ballooning segments in TTS-related VSP exhibit acute functional impairment that is fundamentally reversible, and even when myocardial necrosis occurs, it is considered to be focal in nature. Therefore, recovery of myocardial function in the ballooning segments can be anticipated, suggesting that the long-term prognosis of TTS-related VSP may be more favorable than that of post-ACS VSP. However, reports of VSP associated with TTS are limited, and among the 24 reported cases excluding the present case, the longest reported follow-up period is 16 months. To date, no case has documented long-term postoperative follow-up extending to 10 years, as achieved in the present case. Further accumulation of cases is therefore required to clarify the prognosis and to establish optimal treatment protocols.

The rarity of VSP associated with TTS has made it difficult to elucidate the mechanisms underlying the development of VSP in TTS. We believe that myocardial fragility caused by focal myocardial necrosis and increased systolic wall stress resulting from apical ballooning are the main contributing factors. Histopathological examination of the myocardium in cases of VSP or left ventricular rupture associated with TTS reveals infiltration of lymphocytes and macrophages, loss of myocardial cells, myocardial necrosis, myocardial fibrosis, and CBN.^7–9)^ In this case, histopathological examination of the ventricular septum revealed CBN along with focal myocardial drop-out and fibrosis. The CBN, characterized by hypercontraction of sarcomeres and dense eosinophilic transverse bands, occurs in conditions of increased catecholamine concentration or following reperfusion after myocardial ischemia, and is also observed in TTS along with myocytosis.^2,10–12)^ Grassi et al.^13)^ reported that 10 out of 11 cases of TTS with LV rupture exhibited the CBN. In view of these histopathological findings, it is plausible that in TTS, some myocardial regions undergo necrosis, become structurally fragile, and eventually rupture. In TTS, global LV function generally recovers, suggesting that the myocardial necrosis observed in TTS is likely focal, confined to limited segments rather than involving the entire ballooning area. With respect to LV wall tension, in a normal heart, the LV dimension decreases and the LV wall thickens during systole. However, the ballooning segments of the LV in TTS fail to contract during systole, preventing normal reduction of the LV radius, and either fail to exhibit normal systolic thickening or become thinned, neither of which involves wall thickening. According to Laplace’s law, these result in increased wall tension in the ballooning segments of the LV wall; this systolic increase in wall tension in the ballooning segments has not been previously described. The necrotic areas of the ventricular septum are fragile, and the added increase in wall tension can lead to rupture. If LVOTO is present, the intraventricular pressure further increases, exacerbating the wall tension. Therefore, afterload reduction is considered crucial in preventing the development of VSP or LV rupture, as it helps to mitigate the increased wall tension that contributes to the risk of rupture.

The pathogenesis of TTS is not fully elucidated, but it is thought to be triggered by an endogenous adrenergic surge induced by psychological or physical stress.^3)^ Therefore, during the onset of heart failure, the use of catecholamines and/or beta2-agonists should be avoided as they may further exacerbate contractile dysfunction; instead, mechanical support such as IABP, Impella (Abiomed, Danvers, MA, USA), or extracorporeal membrane oxygenation is recommended.^3)^ During the acute phase of VSP, the myocardium of the ventricular septum is extremely fragile, and patch closure carries a risk of residual shunting. Unlike VSP following ACS, recovery of LV function can be expected in VSP associated with TTS. If mechanical support and medical treatment stabilize the hemodynamics, delaying surgery allows not only for recovery of LV function and organ dysfunction^14)^ but also for fibrosis and strengthening of the septal tissue, thereby reducing the risk of residual shunt postoperatively. It should be noted, however, that IABP may worsen LVOTO and systolic anterior motion of the mitral valve if present. In addition, severe arrhythmias should be carefully monitored.^15)^ In this case, the VSP likely developed 3–5 days after the onset of TTS. Hemodynamics were stabilized by IABP and medical treatment, and LV wall motion abnormalities resolved within a month. Surgery was performed on day 37, with an uneventful postoperative course and no residual shunt. This case represents our first surgical experience with VSP associated with TTS, with the operation performed via an LV incision, as is done in post-ACS VSP. Given the potential for recovery of LV function in TTS, we now consider a right ventricular incision approach to be preferable.

## CONCLUSIONS

While VSP is a potentially lethal complication of TTS, recovery of LV function can be anticipated, suggesting that surgical treatment could be deferred, provided that hemodynamic stability is maintained. This case demonstrates that focal myocardial necrosis can occur in TTS. Furthermore, increased wall tension occurs in the ballooning segments during systole, as the LV radius increases and the ventricular wall fails to thicken, in accordance with Laplace’s law. Both myocardial fragility resulting from focal myocardial necrosis and increased LV wall tension due to systolic ballooning are considered to be key mechanisms underlying the development of VSP in TTS.

## DECLARATIONS

### Availability of data and materials

Data are available on request to the corresponding author.
